# Elesclomol, a copper-transporting therapeutic agent targeting mitochondria: from discovery to its novel applications

**DOI:** 10.1186/s12967-023-04533-5

**Published:** 2023-10-20

**Authors:** Mojtaba Tarin, Maryam Babaie, Hossein Eshghi, Maryam M. Matin, Amir Sh. Saljooghi

**Affiliations:** 1 Department of Chemistry, Faculty of Science, Ferdowsi University of Mashhad, Mashhad, Iran; 2 Department of Biology, Faculty of Science, Ferdowsi University of Mashhad, Mashhad, Iran; 3 Novel Diagnostics and Therapeutics Research Group, Institute of Biotechnology, Ferdowsi University of Mashhad, Mashhad, Iran

**Keywords:** Elesclomol, Oxidative stress, Cuproptosis, Cancer, Drug delivery

## Abstract

Copper (Cu) is an essential element that is involved in a variety of biochemical processes. Both deficiency and accumulation of Cu are associated with various diseases; and a high amount of accumulated Cu in cells can be fatal. The production of reactive oxygen species (ROS), oxidative stress, and cuproptosis are among the proposed mechanisms of copper toxicity at high concentrations. Elesclomol (ELC) is a mitochondrion-targeting agent discovered for the treatment of solid tumors. In this review, we summarize the synthesis of this drug, its mechanisms of action, and the current status of its applications in the treatment of various diseases such as cancer, tuberculosis, SARS-CoV-2 infection, and other copper-associated disorders. We also provide some detailed information about future directions to improve its clinical performance.

## Introduction

Copper (Cu) is known as a cofactor for several enzymes in aerobic organisms. This element is essential for a variety of biochemical processes such as eradication of free radicals, generation of mitochondrial energy, and iron homeostasis [1]. Despite its crucial role in a variety of biochemical processes, Cu deficiency or accumulation is associated with various diseases, including cancer, cardiovascular diseases, Wilson’s disease (WD), Menkes disease (MD), and neurodegenerative disorders [2]. Copper toxicity can give rise to various problems, acting via different mechanisms including oxidative damage, modification of gene expression, and impairment of mitochondrial function [3]. The excess of copper can alter the delicate balance of copper homeostasis, which is rigorously controlled to avoid toxicity [4]. Notably, both severe copper overload (as seen in Wilson's disease) and copper deficiency (in Menkes disease) are linked to specific disorders. Moreover, disturbances in copper homeostasis have been identified in neurodegenerative conditions such as Alzheimer's, Parkinson's, and Huntington's disease [5]. The repercussions of copper toxicity also extend to genetics, with resistance to copper toxicity governed by allelic and expression variations at multiple genetic sites [6]. Furthermore, exposure to copper can induce changes in neuropeptides, affecting the signaling pathways that are crucial in responding to heavy metal exposure [7]. In summary, elevated copper levels could wield toxic effects through various mechanisms, leading to a variety of diseases and perturbations in cellular functions. Various hypotheses such as the induction of reactive oxygen species (ROS), caspase-independent cell death, induction of apoptosis, and inhibition of the ubiquitin proteasome system have been suggested to explain why high levels of copper are toxic to cells [8, 9]. Recently, cuproptosis has been described as a novel cell death pathway, in which copper can directly bind to proteins of the lipoylated TCA cycle [10]. To avoid copper toxicity, a regulated network of proteins is developed to control copper levels in cells by transporting, escorting and storing copper. Hence, disruption of the copper-regulating network could be fatal [11]. In light of these facts, this strategy could be invoked as a prominent strategy to combat copper-associated diseases.

Elesclomol (ELC) is a mitochondrion-targeting copper ionophore developed as a chemotherapeutic agent. This bis(thiohydrazide) amide binds to copper(II) in a 1:1 ratio [12] in the extracellular environment, generating a membrane-permeable complex that can enter and transport copper to mitochondria. After the reduction of copper(II), copper(I) is released in mitochondria [13]. The current review is focused on the discovery, synthesis, mechanisms of action, medical applications, and advances in delivery methods of ELC. We aim to provide a portrayal of ELC as a copper-transporting therapeutic agent, which could guide researchers for more intelligent applications of this drug.

## Discovery of ELC

**Scheme 1:**
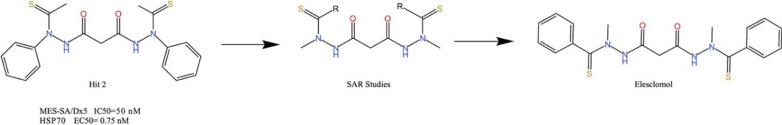
The discovery of elesclomol N'1, N'3-dialkyl-N'1, N'3-di-(alkylcar‐bonothioyl) malonohydrazide (ELC)

Synta Pharmaceuticals company has introduced the ELC (STA-4783; N'1, N'3-methanethiol-N'1, N'3-di (phenylcarbonothioyl) malonohydrazide) **1**—as an anticancer drug for breast cancer, non-small cell lung cancer, and sarcoma [14, 15]. Phenotype screening of small molecules was employed to discover ELC as an anti-cancer moiety. The discovery of ELC was initiated by screening Synta’s unique compound library using cytotoxicity assessment and HSP70 induction assays in a multidrug resistant (MDR) cancer cell line (MES-SA/Dx5) [12]. These investigations led to a hit molecule known as N'1, N'3-methanethiol-N'1, and N'3 diphenylmalonohydrazide **2**. Then, compound **2** was optimized on the basis of the structure–activity relationship (SAR) approach to generate ELC as a novel anticancer agent. Compound **2** was able to induce HSP70 (EC_50_ = 0.75 µM) and indicated strong anti-proliferative activity against the MES-SA/Dx5 cancer cell line (IC_50_ = 50 nM). However, direct exposure to air could easily oxidize two sulphur atoms of compound **2** and consequently inactivate the molecule with oxygen moieties. The metabolically unstable nature of compound **2** necessitated further optimization. SAR studies were used to design more potent inhibitors with better properties. In this regard, various derivatives of the lead compound **2** were synthesized and biologically evaluated to reach ELC **1** (Scheme 1) [12].

## Synthesis of ELC

**Scheme 2:**
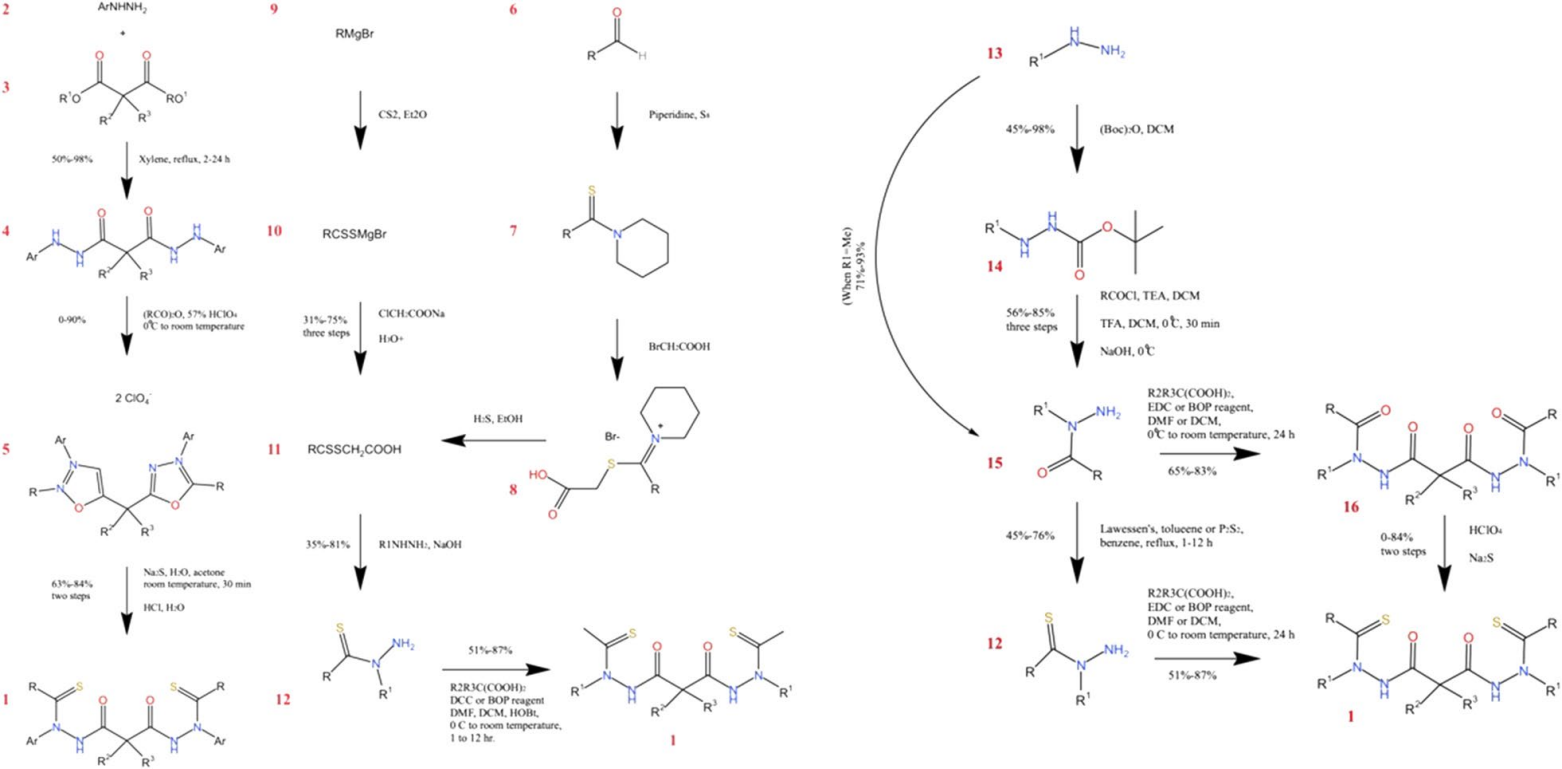
Different synthetic pathways of ELC with different starting materials

Various synthetic pathways beginning with different starting materials were proposed by Chen et al. [12] (Scheme 2) for the preparation of compound **2** derivatives in lead optimization. The advantages and limitations of each synthetic pathway are discussed in this section (Table 1). As illustrated in Fig. 2, the reaction between arylhydrazine (compound **3**) and dialkyl malonate (compound **4**) (at an increased temperature in xylene) gave the corresponding malonyl hydrazide (compound **5**), with a high yield in pathway I, which was acylated with anhydride. Further cyclization using hydroperchloric acid has furnished the compound 6. The following thiolation with acidic hydrolysis and sodium sulfide, generated the final product **1** with the desired yield. However, finding a more general synthetic method seemed to be required, due to several limitations such as the low participation ability of aliphatic anhydrides and the low yield of aliphatic hydrazine-containing derivatives. In the second synthetic method (pathway II), the key intermediate **7** (compound **7**) was synthesized according to the procedure introduced by Jensen and Petersen (1961) [16] from an aldehyde (compound **8**) or Grignard reagent (compound **9**). The reaction between compound **7** and malonic acid using dicyclohexylcarbodiimide (DCC) or benzotriazol-l-yl-oxy-tris-(dimethylamino) phosphonium hexafluorophosphate (BOP) resulted in the production of the final product **1** with a high yield. To synthesize intermediate **7**, the reaction occurred between an aldehyde (compound **8**) and sulfur in piperidine to give thioamide (compound **10**). Treatment of compound **10** with bromoacetic acid generated 1-(1-((carboxymethyl) thio) alkylidene) piperidin-1-ium bromide (compound **11**) in a high yield. Ultimately, the key intermediate **7** could be produced by the treatment of compound **11** with hydrogen sulfide in ethanol. Alternatively, the third pathway could start with the reaction between the Grignard reagent (compound **9**) and carbon disulfide in ether bromide, which could produce the alkanedithioate (compound **12**). This compound led to high yield production of compound **1** after treatment with sodium chloroacetate followed by hydrolysis. These synthetic pathways support each other. For instance, the second pathway is suitable for derivatives that are reactive to Grignard reagent including cyano or carboxylic acid or ester. Alternatively, the third pathway indicates some advantages when the corresponding aldehyde is not very stable or not readily available. A more general synthetic method was also developed (pathway IV) for production of compound **1**. In this pathway, treatment of methylhydrazine (compound **13**) with an acyl chloride or a carboxylic acid resulted in the desired and high-yield production of 2-acylhydrazide (compound **15**). Compound **15** was then selectively acylated to produce **17**, which was converted to compound **1** [17]. Despite the advantages of this pathway, due to the utilization of more steric hindered hydrazine, the primary amino group should be protected by a Boc group [18] for acylation at the 2-position.

**Table 1:** Comparison of different synthetic pathways of ELC

Pathway number	Limitations or advantages	Overall yield (%)
Pathway I	Limited commercial sources	56–91
	Low participation of aliphatic anhydrides	
	Low yield of aliphatic hydrazines	
Pathway II	Suitable for carboxylic acid, cyano, or ester substituted derivatives	42–82
Pathway III	Suitable when aldehyde was not very stable or not readily available	43–84
Pathway IV	Needs protection for sterically hindered hydrazine	32–79
Pathway V	Avoids using explosive perchloric acid, DCC or EDC	60–70
	Column-free isolation of silica gel	

**Scheme 3:**
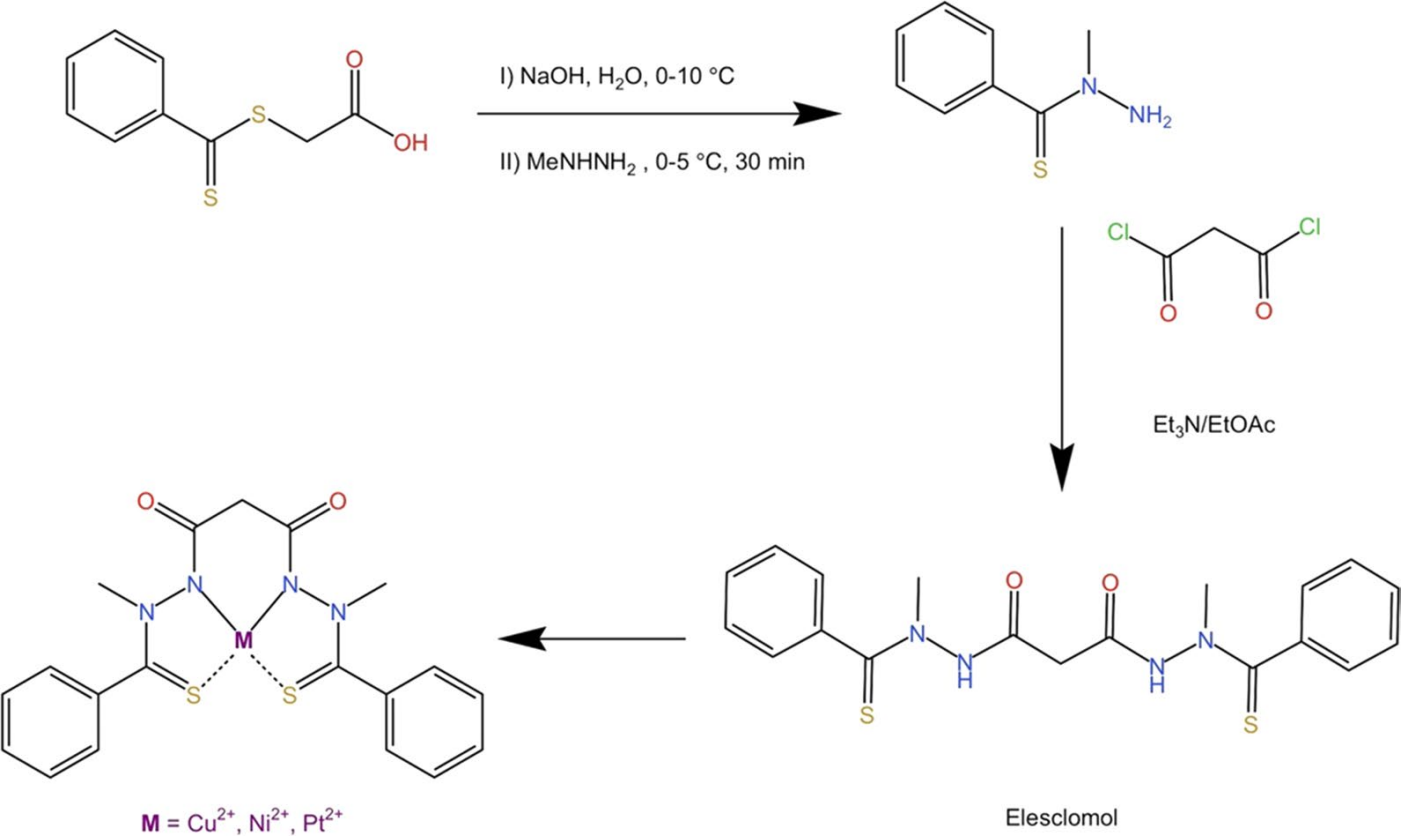
Reaction scheme for the synthesis of ELC and intermediate compounds for the preparation of its complexes with Cu^2+^, Ni^2+^, and Pt^2+^

Encouraged by this progress in the synthesis of ELC, Chen et al. [12] have introduced a convenient method for the synthesis of ELC and its copper complex. Compared to previously reported synthetic methods [12], this new procedure (pathway V) was suitable for current good manufacturing practice (cGMP) of ELC. The advantage of this pathway was the consumption of a class 3 solvent (EtOAc) and easy purification. Moreover, this method has avoided explosive perchloric acid or coupling agents such as 1-ethyl-3-(3-dimethyl aminopropyl) carbodiimide (EDC) or DCC. As exhibited in Fig. 3, direct acylation of *N*-methyl benzothiohydrazide (compound **18**) with malonyl chloride in EtOAc resulted in the production of ELC **1** as a yellow crystalline solid with an isolation yield of 60–70%. Moreover, the copper(II) complex (compound **19**), was synthesized via the reaction between compound **1** and copper sulfate pentahydrate in aqueous acetone at room temperature. It should be noted that other copper(II) salts including copper(II) chloride, copper(II) sulfate hexahydrate, and copper(II) bromide were synthesized using this procedure (Scheme 3). Subsequently, nickel(II) and platinum(II) complexes of ELC have been synthesized and then characterized [12].

The synthesis and structural attributes of elesclomol, along with its interaction strength with copper(II) within the extracellular milieu, have been subjects of scrutiny in various investigations. Zong et al. conducted the synthesis and characterization of a mononuclear complex labeled Cu(L1)2·CH_3_OH·H_2_O, in which L1 signifies 3,5-dibromo-L-tyrosine [15]. Chen et al. undertook the synthesis and characterization of a copper(II) complex, denoted as [Cu(HL)2]·0.5DMF, with HL representing a Schiff N-allylamine-4-(ethylenediamine-5-methylsalicylidene)-1,8-naphthalimide ligand [16]. Jain et al. presented findings on the binding affinity between human serum albumin and three distinct copper(II) complexes, encompassing [Cu(L1)2(H_2_O)2], where L1 designates 2-hydroxy-3-methoxybenzylidene-4H-1,2,4-triazol-4-amine [17]. Min et al. carried out the synthesis and characterization of a copper-bispyridylpyrrolide complex, identified as [Cu(PDPH)Cl], where PDPH represents 2,5-bis(2′-pyridyl)pyrrole [18]. Iqbal et al. undertook the preparation and characterization of a dinuclear centrosymmetric copper(II) complex, linked by para-fluorophenyl acetate ligands [19]. These various studies contribute valuable insights into the synthesis, structure, and the affinity with which ELC interacts with copper(II) within diverse environments.

## ELC mechanism of action: from in vitro studies to clinical trials

ELC operates as a copper ionophore, which targets mitochondria by creating a complex with copper (Cu) and its transport into the mitochondria [19, 20]. Within the mitochondria, ELC-Cu(II) reduces to Cu(I), which triggers the generation of ROS [21]. ELC incites oxidative stress by elevation of ROS levels beyond the sustainable thresholds in cancer cells. This is accomplished by augmentation of ROS production and induction of a transcriptional gene profile that mirrors an oxidative stress response [22]. Previous studies have demonstrated the ELC capacity to elevate ROS production and provoke a dose-dependent inhibition of mitochondrial NADH-ubiquinone oxidoreductase activity [13]. The escalated ROS levels and resulting cytotoxicity prompted by ELC could arise from the uncoupling of mitochondrial oxidative phosphorylation and/or the inhibition of electron transport activity. Notably, the ability of ELC to specifically induce mitochondrial ROS production stands out as a distinct property of this compound.

### Primary view on ELC mechanism of action

Numerous studies have been conducted to elucidate the mechanism of action for anti-cancer activity of ELC. This molecule was found as an apoptosis stimulant with potent pro-apoptotic activity. ELC also exhibited strong anti-cancer effects against various tumor cells, particularly several MDR tumor cell lines that were resistant to Taxol (paclitaxel). Metals are widely distributed in nature and are essential to many facets of biological activities. They are required for both structure and activity of some proteins. Metal importers, metal exporters, and metal stores regulate the amount of metal that is available in the cytoplasm. Both physiological and pathological processes require copper, and its improper metabolism has been linked to several illnesses, particularly cancer [23–25]. Since copper trafficking is preferentially regulated in cancer cells, it has been demonstrated that the blockade of copper trafficking by pharmacological inhibition of copper chaperones can treat a variety of cancers. However, due to its potential cytotoxicity, the concentration and transport of intracellular copper must be carefully managed [26]. ELC monotherapy and its combination with other anti-cancer agents have already been practiced. The free acid form of ELC acts as an active substance and binds to copper ions (Cu^2+^), which are present in the serum. Unlike free ELC, this complex is efficiently adsorbed by cancer cells. The copper present in the complex undergoes the reduction reaction (Cu(II) to Cu(I)) inside the cell, leading to disruption of oxidative phosphorylation in mitochondria, where it can induce oxidative stress through the accumulation of ROS. Formation of the ELC-Cu^2+^ complex and reduction of Cu^2+^ could generate damaging ROS. On the other hand, the generated Cu^+^ could react with O_2_ to produce superoxide, which may produce damaging H_2_O_2_ [27]. Moreover, the highly reactive hydroxyl radical can be produced from the reaction between H_2_O_2_ and Cu^+^ [27, 28]. ELC-Cu complex can be deployed from the extracellular environment to the intracellular compartments. Following the uncoupling of copper from the complex, ELC can be effluxed from the cells to transport copper again. This process can lead to accumulated copper in mitochondria, which is correlated with cytotoxic activity. In other words, the reduction of the copper initiates mitochondrial induction of ROS. This mechanism of action for the anti-cancer activity of ELC has been supported by various studies. Vo et al. have shown that the reversible one-electron reduction could take place in the ELC-Cu complex at biologically accessible potentials, while the free ELC is electrochemically inactive [29]. Furthermore, under physiological conditions, the conditional stability constant for ELC-copper(II) bonding is 24-fold higher than TRIEN (a strong copper(II) chelator). NCI COMPARE analysis revealed that the action mechanism of ELC-Cu(II) resembles other cytotoxic copper chelating compounds for cytotoxicity. Highly specific copper chelators such as triethylenetetramine and tetrathiomolybdate significantly decreased the cytotoxic effects of both ELC and ELC-Cu(II) complexes on K562 cells. This property indicates the significance of copper in the cytotoxicity of ELC. Hence, the authors concluded that ELC activity is dependent on a redox-active metal. The ability of redox-inactive metal complexes to suppress cell growth remains controversial. To address this question, Yadav et al*.* investigated the effects of nickel(II)-ELC and platinum(II)-ELC complexes on human leukemia K562 cells. Their findings indicated that nickel(II)-ELC and platinum(II)-ELC complexes were 34 and 1040 times weaker than the Cu(II)-ELC, respectively. Thus, a redox-active metal is necessary for the inhibitory effects of ELC, and cell growth inhibition of ELC-Cu(II) complex is exerted via generating copper-mediated oxidative stress [28].

ELC has been reported to inhibit tumor-cell proliferation in MCF-7, SK-MEL-5, and HL-60 cells with IC_50_ values of 110, 24, and 9 nM, respectively [30]. It was also shown that ELC could induce tumor-cell apoptosis via ROS production in various cancer cell types [31]. In vitro results have indicated that ELC could regulate the expression levels of heat shock stress response and metallothionein genes; for instance, ELC (100 nM) increased the *HSP70* transcript levels of Ramos Burkitt's lymphoma B cells in a time-dependent manner.

Different forms of ELC have been synthesized. Among them, the copper(II)-ELC complex was the most active form in human leukemia K562 cells [32, 33]. ELC or ELC-Cu(II) treatment on human K562 cells led to cell death after several hours. This treatment induced double-strand breaks of DNA, and apoptosis with subtle outcomes on the mitochondrial membrane potential. A G1 cell cycle arrest in synchronized Chinese hamster ovary (CHO) cells was observed following the ELC or ELC-Cu(II) treatment. Although they managed to inhibit (weakly) the DNA topoisomerase I, ELC, or ELC-Cu(II) were not effective on DNA topoisomerase II. ELC or ELC-Cu(II) treatments have been investigated on cross-resistant cell lines, which overexpress several ATP-binding cassette (ABC) type efflux transporters. The obtained results demonstrated that none of the ELC or ELC-Cu(II) treatments were effective on cells overexpressing either ABCG2 (BCRP) or ABCB1 (Pgp), while the cells overexpressing the ABCC1 (MRP1) were rather cross-resistant [32]. Moreover, computational calculations have supported the use of ELC as an effective drug to treat prostate cancer [34].

Increased metabolism in cancer cells results in elevated levels of superoxide and ROS in mitochondria [35]. The produced hydrogen peroxide and increased ROS levels [36–39] could result in aggressive tumor growth [35, 38]. Disrupting the equilibrium concentration of ROS is known as an effective anticancer strategy [36, 39, 40] and the increased ROS levels could reduce the antioxidant activity of cancer cells compared to normal cells. Respiratory chain reactions of mitochondria are attributed to be the main source of ROS. ELC partly exerts its effects by disrupting the mitochondrial metabolism of cancer cells to induce apoptosis. Mitochondrion as a promising target for cancer chemotherapy significantly contributes to cell death and survival [41]. It acts as a critical component of redox control in cancer cells and is the main producer of energy [adenosine triphosphate (ATP)] and ROS within the cells [13, 42, 43]. Using antioxidants N-acetylcysteine (NAC) and Tiron pretreatment could completely block the ELC-mediated elevation of apoptosis via induction of oxidative stress [31]. This property is an evidence for the pro-apoptotic activity of ELC via ROS generation. It has been revealed that Nucleus accumbens-1 (NAC1) silencing, which mediates ROS production and protects cancer cells from apoptosis in hypoxia, promotes the antitumor efficacy of ELC [44]. To delineate the therapeutic capacity of ELC as an anticancer agent, Modica-Napolitano and Weissig investigated the effects of ELC on the bioenergetics function of mitochondria in an isolated population of mammalian mitochondria. Their results showed that ELC (at a high dose) could uncouple oxidative phosphorylation and inhibit the activity of electron transport in isolated and intact mitochondria. Moreover, mitochondrial NADH-ubiquinone oxidoreductase activity could be inhibited using ELC in freeze-thawed mitochondrial populations in a dose-dependent manner. Accordingly, the ELC-mediated increase in ROS generation and cytotoxicity may somehow be rooted in the uncoupling of mitochondrial oxidative phosphorylation and/or repression of electron transport activity. Since the structure and function of mitochondria differ in normal and cancer cells (e.g., regarding response to uncoupling, membrane permeability, and complex I structure and function), the selective ELC-mediated cytotoxicity on cancer cells could be logical [20]. The mitochondrial selectivity of ELC is not similar to other chelators, such as disulfiram. It could be concluded that ELC interacts with electron transport chain (ETC) to produce high levels of ROS inside the organelle and subsequently leads to cell death [45]. These outcomes point to a unique mechanism of action with significant implications for an amenable cancer therapy approach.

### Cuproptosis, a novel discovered cell death mechanism for ELC

**Fig. 1:**
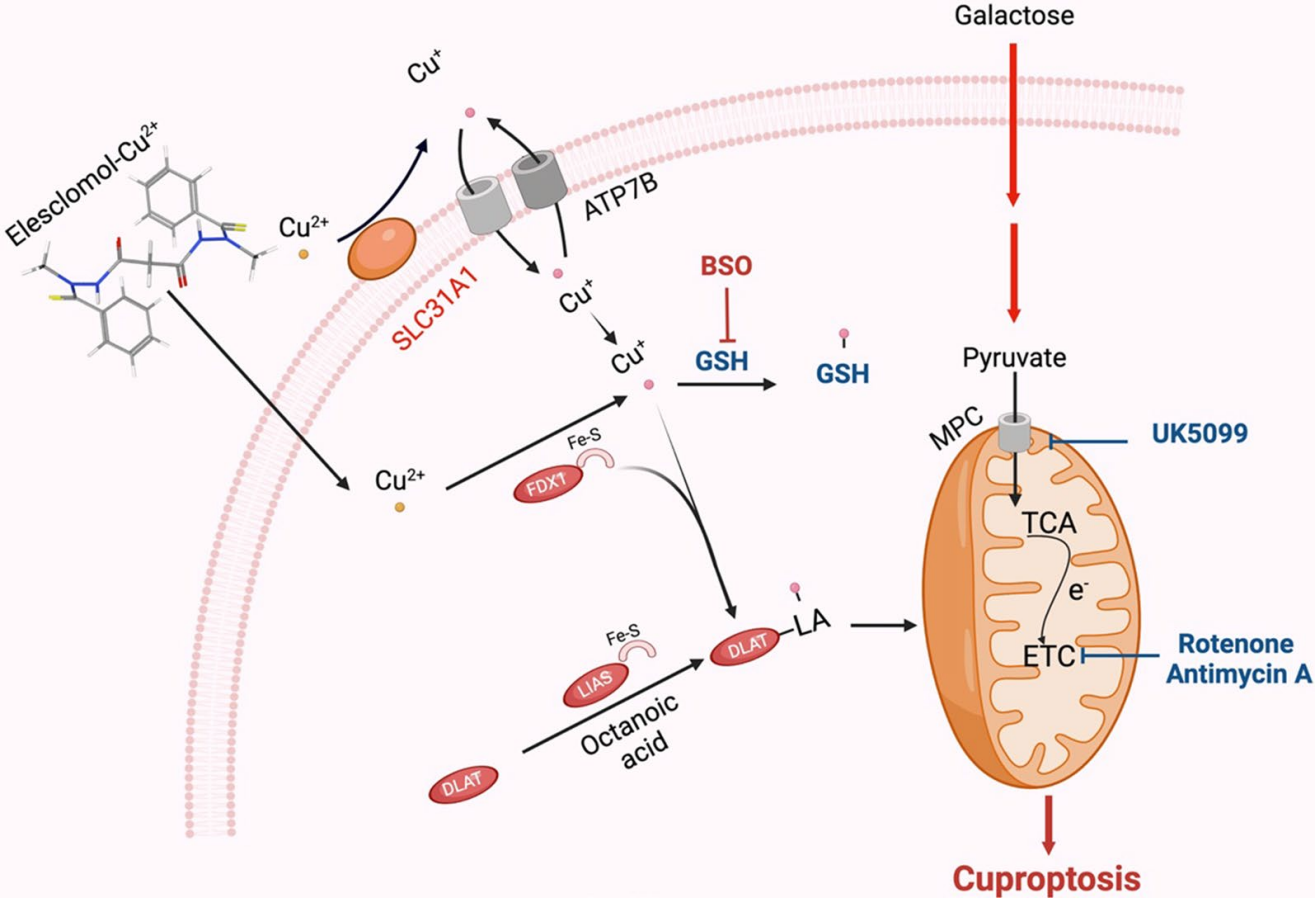
ELC transfers copper (Cu^2+^) from the extracellular matrix to intracellular compartments by binding to it. Cuproptosis is primarily brought on by increased Cu buildup and mitochondrial proteotoxic stress, which is mediated by FDX1. On one hand, FDX1 facilitates the lipoylation (LA) and aggregation of enzymes (particularly DLAT) involved in the control of mitochondrial TCA cycle by reducing Cu^2+^ to Cu^+^. On the other hand, FDX1 facilitates lipoylation and aggregation of enzymes that control the mitochondrial TCA cycle (particularly DLAT) by reducing Cu^2+^ to Cu^+^

Heavy metal ions are essential micronutrients and their insufficiency or excessive abundance can trigger cell death. A novel form of regulated cell death (RCD) has been identified, known as "cuproptosis", which is different from oxidative stress-related cell death. In a recent study, Tsvetkov et al. [10] unveiled cuproptosis as a copper-dependent non-apoptotic cell death pathway [9, 10]. In this distinct pathway, copper directly binds to the tricarboxylic acid (TCA) cycle's lipoylated components. Subsequent aggregation of these copper-bound proteins and disruption of Fe-S proteins induce proteotoxic stress and result in cuproptosis [2, 9, 10] (Fig. 1). In other words, excess copper levels can induce cell death by binding to lipoylated components of the TCA cycle, which results in protein aggregation and proteotoxic stress. These facts may explain the need for ancient copper homeostatic mechanisms [10]. A study conducted by Chu and colleagues provides partial insights to further comprehend the molecular mechanisms of cuproptosis in clear cell renal cell carcinoma** (**ccRCC) and could be helpful for the development of personalized therapeutic strategies targeting copper or cuproptosis [46].

Tang et al. examined whether the toxicity of Cu ionophores, especially ELC, depended on the induction of known cell death modalities. They found that the hydrophilic antioxidant glutathione (GSH) blocked the toxicity of ELC-Cu by chelation of intracellular Cu [47]. It was shown that cells relying on galactose-mediated mitochondrial respiration are more sensitive to ELC-Cu-induced growth inhibition than cells that rely on glucose-induced glycolysis. Furthermore, hypoxia (1% O_2_) reduced cuproptosis sensitivity. The authors identified two mitochondrial proteotoxic stress pathways that mediate cuproptosis using a genome-wide CRISPR/Cas9 knockout screen combined with metabolic and biochemical assays. Cu increases mitochondrial protein lipoylation, which promotes the disulfide bond-dependent aggregation of lipoylated DLAT and subsequent cuproptotic cell death. The authors investigated whether natural Cu stress (without ELC) would induce a similar phenomenon as ELC-Cu [48]. They found that the overexpression of SLC31A1 in cells and the depletion of GSH by BSO could increase susceptibility to cuproptosis. The present findings reinforce the notion that mitochondria are multifaceted regulators of cell death [49], including copper-induced cell death [50]. It also challenges the conventional view that oxidative stress is a fundamental molecular mechanism of metal-induced toxicity [51]. The process and consequences of cuproptotic death deserve further studies.

Cu importers (such as SLC31A1) and exporters (such as ATP7B) also influence intracellular Cu^+^ levels to control cuproptosis sensitivity in addition to Cu ionophores. While BSO encourages cuproptosis by depleting GSH, GSH acts as a copper chelator that abolishes the process since it contains thiols. The electron transport chain complex I/III inhibitors (such as rotenone and antimycin A) and the mitochondrial pyruvate carrier (MPC) inhibitors (such as UK5099) could decrease the effects of ELC on cuproptosis (dihydrolipoamide branched chain transacylase E2 (DBT), glycine cleavage system protein H (GCSH), dihydrolipoamide S-succinyltransferase (DLST), dihydrolipoamide S-acetyltransferase (DLAT)), tricarboxylic acid (TCA) cycle).

#### ELC and ferroptosis

**Fig. 2:**
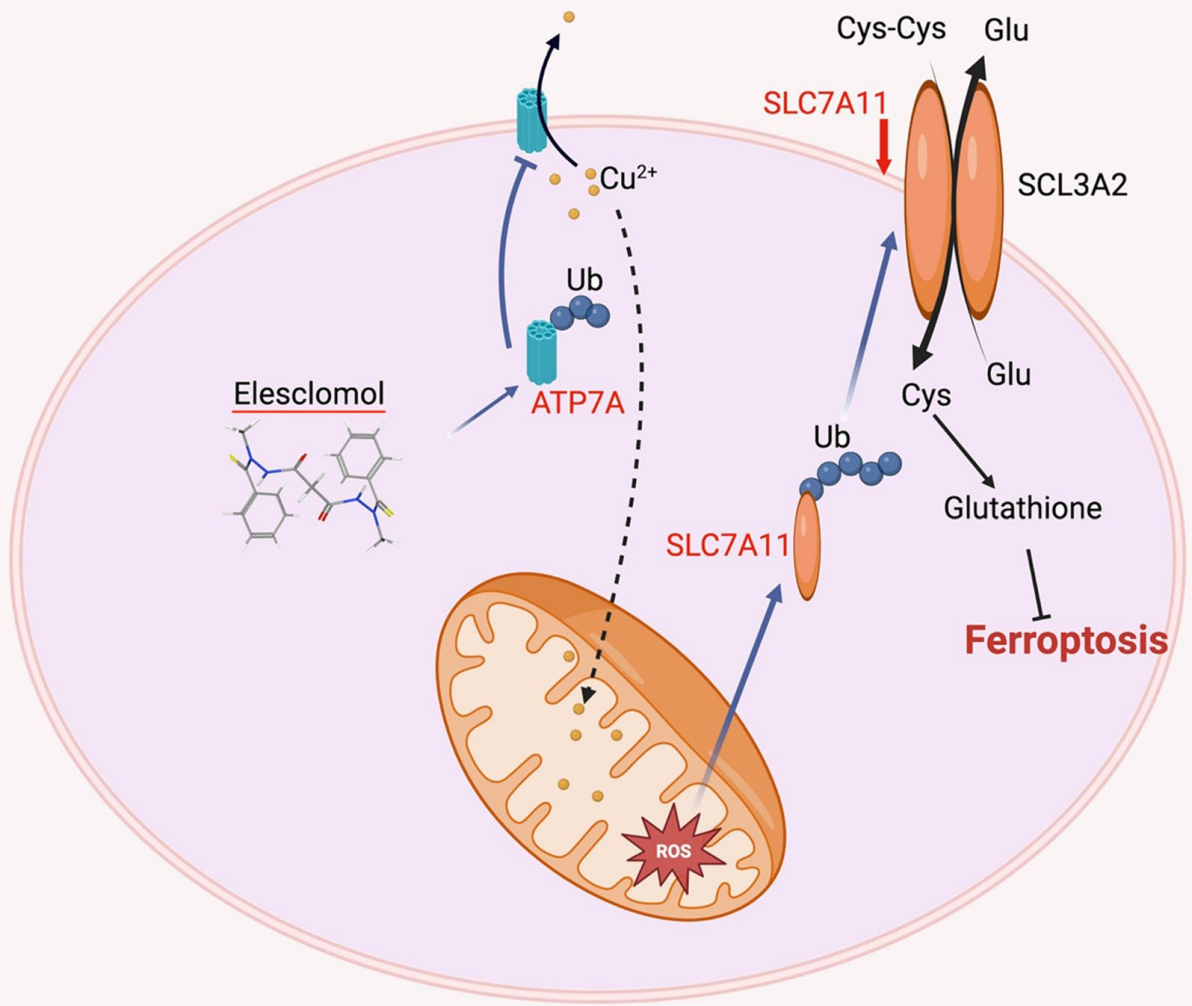
Working model of CRC ferroptosis and ATP7A degradation caused by ELC. ELC increases the concentration of Cu^2+^ in mitochondria and lowers the expression of ATP7A, which causes Cu^2+^ to be retained inside cells and subsequently accumulate ROS. This action encourages SLC7A11 degradation, which increases oxidative stress and finally causes ferroptosis in CRC cells

ELC outperformed some other copper chelators in the challenge of hydrogen peroxide-induced oxidative stress and effectively inhibited the growth of colorectal cancer cells. This cytoprotective property of copper may be connected to the antioxidant role of superoxide dismutase 1. ELC induces significant cell death in colorectal cancer cells (CRC) in the presence of exogenous copper, which can be partially restored by administration of N-acetyl-L-cysteine. However, no evident DNA damage or cleaved caspase-3 was seen. This observation indicates that the cell death brought on by ELC may not be due to apoptosis. Treatment with ELC had no obvious toxicity to several other normal organs of the subcutaneous xenograft mouse model and suppressed colorectal tumor growth. ELC decreased the concentration of copper in the media while increasing the concentration in the cytoplasm and mitochondria. This means that ELC transports copper from the medium into mitochondria. Additionally, ELC reduced the expression of ATP7A and ATP7B (copper‐transporting ATPase 1), which are essential for copper retention and ELC's antitumor effects. Several datasets revealed that ATP7A is overexpressed in CRC patients and is correlated with poor overall survival and progression-free survival compared to normal tissues [53–58] (Fig. 2).

ELC downregulates ATP7A through a protein degradation route, which was evidenced by the fact that using proteasome inhibitors (such as MG132) could prevent ELC from causing a drop in ATP7A protein level. The probable essential elements that have been suggested to be involved in the degradation of ATP7A were investigated for better examination of the processes underpinning ELC-induced ATP7A degradation. It was suggested that Cav-1 (Caveolin‐1) may not be involved in ELC-induced ATP7A degradation in CRC cells [59]. SLC7A11 (solute carrier family 7 member 11) is overexpressed in malignant tissues relative to normal tissues, and this overexpression is also connected with CRC metastatic recurrence, according to the analysis of various datasets [60–62]. ELC causes the breakdown of ATP7A and SLC7A11, while the exact mechanisms remain to be obscure. ELC reversed the overexpression of exogenous ATP7A and SLC7A11 in CRC cells. It has been reported that ATP7A controls oxidative stress by preventing copper from leaving cells. This prevents ROS production and inhibits the degradation of SLC7A11, which increases the oxidative stress brought on by ELC. CRC could also be suppressed by ELC-mediated degradation of ATP7A, which is an overexpressed protein when ELC and copper are used together as a therapy. Moreover, the effect of various copper chelators on the expression of ATP7A was investigated. ELC, but not other copper chelators, was discovered to activate ATP7A degradation, which causes copper to be retained in cells and causes ferroptosis in CRC cells.

ELC was first used to treat non-small cell lung cancer, melanoma, and sarcoma as an apoptotic promoter [14]. It has been demonstrated to disrupt the cytoskeleton in tumor cells, increase cytotoxicity, and delay growth-free survival [63]. ELC increases ROS, which inhibits the development of cisplatin-resistant lung cancer cells and causes oxidative stress in melanoma and leukemia cells. According to the study by Gao et al., ELC causes ferroptosis in colorectal cancer cells but not apoptosis [64]. Although it has been established that copper and nickel may shield neuronal cells from ferroptosis, it is typically thought to be an iron-related pattern of cell death. Through interactions between copper and iron or by controlling the production of ROS in cells, copper may control ferroptosis. Copper-mediated ferroptosis is largely attributed to lipid hydroperoxide, which could be induced by the Fenton reaction, making copper complexes, and copper chelators potential anticancer agents [65].

It was shown that CTR1 is not involved in the anti-CRC function of ELC and ATP7A degradation is a precondition for ROS buildup and ferroptosis in CRC cells [64]. ATP7A as a copper transporter plays a role in the development of tumors and the resistance of cancer to treatment. Compared to cisplatin-sensitive NSCLC tissue, ATP7A is overexpressed in cisplatin-resistant non-small cell lung cancer tissues. ELC reduces the expression of ATP7A and SLC7A11 in CRC cells, and it also protects SLC7A11 from degradation, which increases oxidative stress and leads to ferroptosis. It was not established whether ATP7A is a specific target of ELC. It has been shown that ELC breaks down ATP7A through the prevention of copper outflow, although it has no apparent effect on Cav-1 expression. Therefore, the identification of E3 ligase for ATP7A will contribute to a better understanding of this process.

### Clinical trials on ELC

Several clinical trials have been conducted on ELC and interpreted based on the old concept of the action mechanism for this drug. It would be impressive if the results were revised and discussed based on cuproptosis. Moreover, the design and operation of new clinical trials based on the novel view are impressive and appreciated [66]. However, the following trials are pointed out based on the old view. The phase I clinical trials on patients with refractory solid tumors indicated that co-treatment with ELC and paclitaxel was well tolerated. This combinatorial therapy has shown a toxicity profile observed in paclitaxel monotherapy [63, 67]. In a phase II clinical trial with a double-blinded (randomized, and controlled) design on patients with stage IV metastatic melanoma, administration of the ELC-Taxol combination led to a prolonged progression-free survival as compared with treatment by Taxol alone [68–71]. Furthermore, the effects of ELC in combination with paclitaxel were examined in patients suffering from metastatic melanoma through a comprehensive phase III clinical trial. The obtained results revealed that, in patients with increased lactate dehydrogenase (LDH) levels, mortality was raised, while progression-free survival was significantly improved in 68% of the population with normal LDH levels [72–74]. LDH converts pyruvate to lactate, which is elevated in tumors via glycolysis for energy production. A high LDH level in the bloodstream is detected when the main mechanism of energy metabolism is changed from mitochondrial respiration (using ETC) to glycolysis. In agreement with the results of a phase III clinical trial on metastatic melanoma [72] the observed LDH dependency suggested that ELC is an effective agent to treat tumors with high mitochondrial respiration [13].

## Applications of ELC

Copper chelating agents have exhibited promising potentials to address copper-related conditions, including diseases such as cancer, tuberculosis, and SARS-CoV-2 infection [75]. These agents effectively restrain copper accumulation in body tissues and bloodstream. Therefore, copper-chelating agents can hamper inflammation and diminish the impact of copper-mediated ROS [76]. Notably, clinical trials have appraised copper-chelating therapies for diseases, which are marked by an excessive elevation of copper levels, including cancer [76]. Within the realm of cancer treatment, copper coordination complexes have been introduced as prospective therapeutic entities, although some recent advancements underscore their role as agents with anticancer attributes [26].

Moreover, it has been reported that ELC can significantly improve the efficacy of therapeutic agents in several diseases such as cancers, Menkes disease, Ewing sarcoma, tuberculosis, and hypertrophic scar formation.

The following sections are devoted to the description of ELC effects on different diseases.

### Cancers

ELC exhibited promising results against cancer in some clinical trials while it was not quite successful in some others. In the old view, ELC was an oxidative stress inducer for cancer suppression [66]. However, it was recently nominated as a cuproptosis (a novel form of cell death) inducer [10, 66]. Mitochondrial metabolism, involved in resistance to drugs and the survival of cancer cells, could be spontaneously enhanced in various cancers, including leukemia, breast cancer, and melanoma [77]. The enhanced metabolism characteristic is a suitable condition for ELC to decrease respiration's spare capacity by the inhibition of TCA cycle components [10]. Although all cancer cells and patients are not sensitive, cancer stem cells, drug-resistant cancer cells, cancer cells with lower glycolytic activity and cancer patients with low serum LDH levels are sensitive to ELC [66]. Enhanced mitochondrial metabolism has been observed in these cancer cells and patients [66]. ELC has shown a higher efficacy compared to traditional chemotherapy drugs in certain contexts. In the context of colorectal cancer (CRC), ELC-induced copper chelation inhibited CRC by targeting ATP7A and regulating ferroptosis [64]. In the context of BRCA1-mutated breast and ovarian cancers, ELC exhibited selective sensitivity to cancer cells with mutant BRCA1 and showed synergistic effects when combined with DNA-damaging agents or PARP inhibitors [78]. In the context of copper metabolism diseases, ELC can restore intracellular copper homeostasis and may have the potential to treat human diseases of copper metabolism [79]. However, it is important to note that the efficacy of ELC may vary depending on the specific cancer type and genetic mutations involved.

In the following sections, some studies on ELC-sensitive cancers are mentioned.

#### Lung cancer

**Fig. 3:**
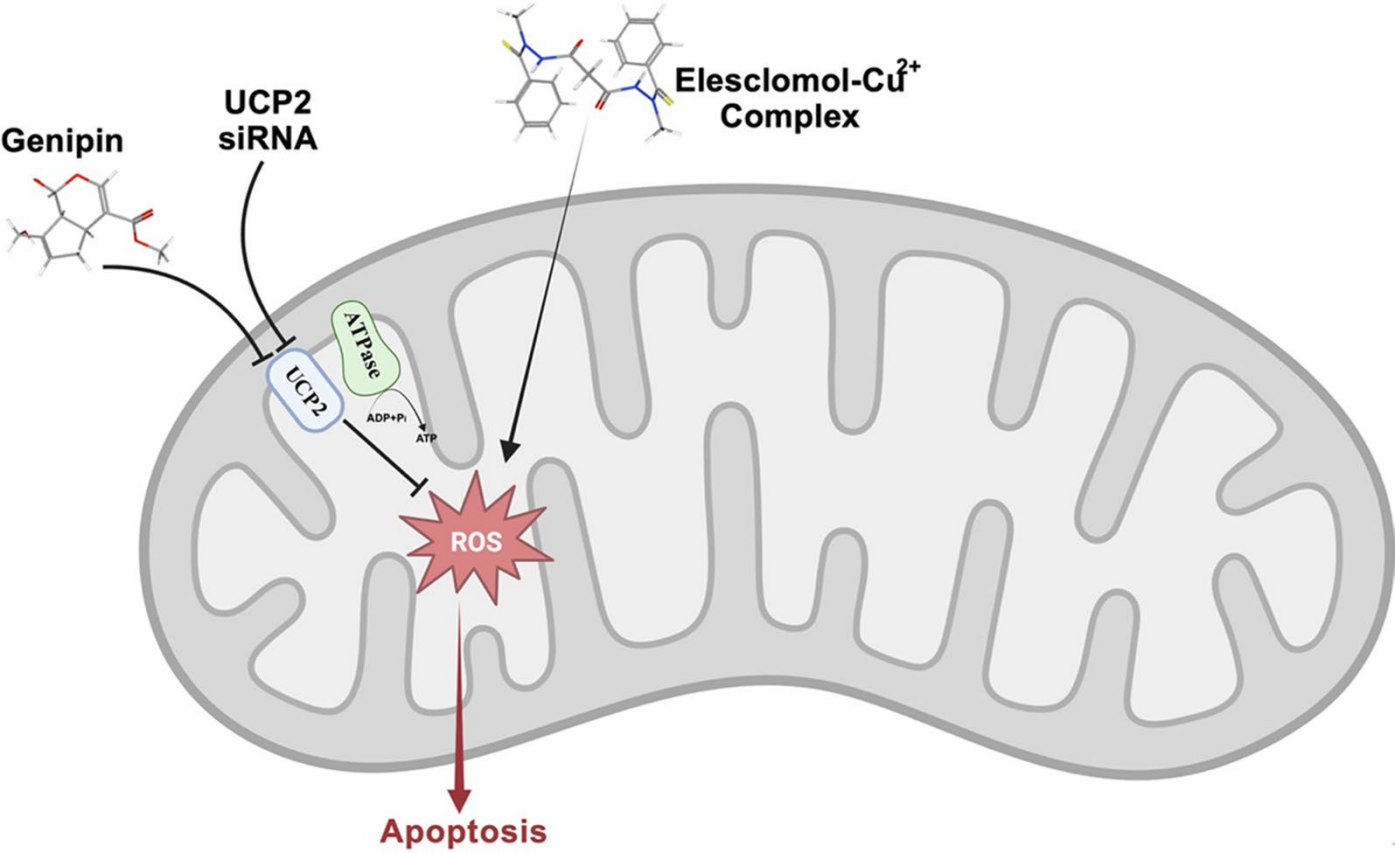
Schematic illustration of the synergistic anticancer effects of ELC and genipin. The ELC-Cu^2+^ complex can enter cancer cells to induce an increase in mitochondrial oxidative stress, which can be reduced by UCP2-mediated uncoupling of oxygen consumption with ATP synthesis. When UCP2 is inhibited with genipin or UCP2 siRNA, apoptosis is triggered and ROS production is augmented

Lung cancer is the most prevalent cancer type worldwide, which occurs due to multiple genetic mutations. The most prevalent genetic alteration in adenocarcinoma patients happens in *KRAS.* Personalized medicine and targeted therapy approaches are still required for the treatment of these cases. Albayrak et al*.* investigated the effects of ELC on Calu‐1 and A549 (as KRAS mutant cell lines) by evaluation of cellular apoptosis, survival, and metastasis. Their results indicated that ELC can alter the expression of apoptotic‐related proteins (CASP‐3, CASP‐9, BCLXL, and BCL2) and inhibit the metastatic‐related proteins (Vimentin, E‐cadherin, MMP‐2, and MMP‐9) in both cell types. The results of the wound healing assay demonstrated that ELC was also effective on cell migration. It was shown to induce total oxidant status (TOS) and malondialdehyde (MDA) in Calu‐1 cells [80]. The cisplatin resistance of lung cancer cells has already been reported, in which metabolic reprogramming and the transformation of glycolysis metabolism to mitochondrial metabolism are at work [81]. Wangpaichitr et al. have used ELC to selectively overcome cisplatin-resistant lung cancer cells. They aimed to elevate the ROS levels for the treatment of patients who failed cisplatin therapy. This strategy was beneficial due to high basal levels of ROS in resistant cells in comparison to normal cells. Hence, increased ROS had toxic effects on cisplatin-resistant cells while sparing normal cells. The application of N-acetylcysteine (NAC) (a ROS neutralizer) has eliminated the cytotoxic effects of ELC. This study confirms that the observed toxicity was attributed to increased ROS [82]. The uncoupling protein-2 (UCP2) can limit the ELC-induced cytotoxicity by attenuation of ROS responses. Upon exposure to ELC, glucose uptake, ROS production, and cell survival of A549 lung cancer cells with high UCP2 expression were partial. However, using *UCP2* knockdown or genipin (a blocking UCP2 agent), the glucose uptake and ROS production were elevated. Therefore, it could be concluded that co-treatment of ELC with genipin in cancers with a high level of UCP2 expression could be considered an effective therapeutic strategy (Fig. 3). In this context, Lee et al. have evaluated the consequences of this co-treatment on A549 cells. Their results indicated a decreased glucose uptake and elevated cellular and mitochondrial ROS generation. They also detected reduced colony-forming capacity, cell survival, and mitochondrial membrane potential. Furthermore, induction of apoptosis was confirmed by annexin V assay, and elevated protein cleavage by PARP and Caspase-3 was observed. An in vivo study using the A549 xenograft mouse model has indicated a markedly suppressed tumor growth in the ELC and genipin co-treatment group compared to control and monotherapy (with ELC or genipin) groups [19].

A recent study showed that ABC1 (a human lung cancer adenocarcinoma cell) is an ELC-sensitive cell in which ELC induces cuproptosis and upregulates TCA-related metabolites (glutamine, α-ketoglutarate, succinate, citric acid, cis-aconitate, and sedoheptulose 7-phosphate) [10]. However, treatment of A549 cells with ELC resulted in no changes in TCA-related metabolites. Basal respiration and the production of ATP were not affected by ELC. Hence, it could be deduced that although the electron transport chain is not directly inhibited by ELC, TCA cycle proteins are involved in this newly discovered mechanism of cell death [10].

#### Breast cancer

Breast cancer is among those cancers, that are highly dependent on mitochondrial metabolism and show spontaneous enhancement of mitochondrial metabolism [66]. Alli and Ford have shown that treatment with ELC could be beneficial for BRCA1-mutated and/or basal-like breast cancers. These breast cancers are partly insensitive to present “targeted” therapy regimens. ELC elevates the ROS levels to exceed the viable threshold, which in turn antagonizes the cellular response to oxidative stress and triggers apoptosis [83]. Moreover, ELC suppresses the growth of human breast cancer epithelial cells without affecting normal breast cells and improves the apoptotic effects of chemotherapy drugs such as doxorubicin or paclitaxel. These functions were the result of the increased levels of cleaved Caspase-3, P27Kip1, and P21Cip1 and decreased levels of NF-κB activity. ELC activates the P38 mitogen-activated protein kinase and c-Jun N-terminal kinase (JNK). Activated JNK1 is responsible for the induction of apoptosis. ELC also induces AKT/HSP70 survival signaling. Suppression of AKT activation through a small-molecule inhibitor has increased ELC-elicited apoptosis through a cellular feedback mechanism. These observations suggest that co-treatment of ELC with chemotherapeutic and AKT-targeting agents can induce apoptosis in breast cancer cells and may be warranted [15].

#### Ovarian cancer

Ovarian cancer is also among those cancers, that are highly dependent on mitochondrial metabolism and show spontaneous enhancement of mitochondrial metabolism [66]. So, it could be ELC-sensitive [66]. Disease recurrence and chemo-resistance in ovarian cancer (OC) is the main cause of mortality, which is attributed to the stem-like tumor-initiating cell (TIC) population. Harrington et al. have analyzed RNA sequencing results of OC cells, grown in TIC conditions. They found significant enrichment of genes contributing to ROS and oxidative phosphorylation pathways. To identify the TICs-targeting drugs, high-throughput drug screening approaches were employed. According to obtained results, disulfiram, ELC, bardoxolone methyl, and salinomycin, were selected for in vitro biological evaluation. ELC was able to induce ROS accumulation and enhance cell death [84]. Moreover, in patients with tubal cancer, platinum-resistant recurrent ovarian cancer, or peritoneal cancer, the combination of ELC with weekly use of paclitaxel resulted in the median overall survival and progression-free survival of 13.3 and 3.6 months, respectively [9, 10, 70].

#### Melanoma

Melanoma is also highly dependent on mitochondrial metabolism and shows spontaneous enhancement of mitochondrial metabolism [66]. As mentioned earlier, ELC has been used for melanoma treatment in phase II clinical trials [42–44]. Based on accumulated data [85], the Achille's heel of melanoma is ROS generation. Intrinsic drug resistance in melanoma cell subpopulations arises from the survival of slow-cycling melanoma cells. Cierlitza et al. have suggested that ELC could overcome multidrug resistance by killing the slow-cycling subpopulations [86]. Corazao-Rozas et al. have also studied the possible effects of mitochondrial oxidative stress against melanoma cells, which were resistant to BRAF-mutant inhibitors. Attained results indicated that ELC could kill vemurafenib-resistant melanoma cells via augmentation of ROS levels [87]. Hence, the combination of BRAF^V600E^ inhibition and ELC could be considered a rational strategy to overcome drug resistance in metastatic melanoma. Dysregulation of ROS generation using ROS inducers has significant advantages in the treatment of metastatic melanoma. Therefore, Wong et al. evaluated the effects of co-treatment of oxidant drugs (such as amino-artemisinin artemisone) and novel prenylated piperazine derivatives with redox or prooxidant drugs like ELC-Cu(II) (as the redox component) on A375 human malignant melanoma cells. The results of this combination therapy indicated reduced cell counts and increased apoptosis [88].

### Infectious diseases

#### Tuberculosis

Tuberculosis (TB) is an airborne infectious disease caused by *Mycobacterium tuberculosis* (Mtb) [89]. Based on the World Health Organization (WHO) report, TB is the second leading killer related to infectious diseases after COVID-19 and the 13th leading cause of death worldwide, responsible for 1.6 million deaths in 2021 (https://www.who.int/news-room/fact-sheets/detail/tuberculosis). Although vaccines are in progress to prevent TB [89], established TB by multidrug-resistant strains needs effective treatments. In humans, Mtb lives in oxidative unfavorable conditions. Hence, oxidant or redox agents could interfere with homeostatic regulation. The ability of ELC to form a redox-active copper chelate has encouraged Ngwane et al. [90] to examine the effects of this anti-cancer drug on Mtb. This study was impressive due to the important role of copper in the elimination of bacterial infection in the innate immune system. They showed that the copper-ELC complex could significantly increase oxidative stress in tumor cells and exert potent inhibitory effects on Mtb H37Rv at a minimum concentration of 10 μM (4 mg/L). It indicated a synergistic interaction with rifampicin and known tuberculosis drugs (such as isoniazid and ethambutol) against MDR clinical isolates of Mtb. Moreover, controlled supplementation of ELC with copper elevated Mtb sensitivity by more than 65 folds. Obtained results have led to ELC repurposing as a potential drug against Mtb [91, 92].

#### COVID-19

A recent pandemic began in late 2019 and introduced the leading killer infectious disease (COVID-19) caused by a novel coronavirus viz respiratory syndrome coronavirus 2 (SARS-CoV-2). Until 16 August 2023, 769,806,130 confirmed human infections and 6,955,497 confirmed deaths have been reported (https://covid19.who.int/). This pandemic continues despite widespread vaccination programs with various available vaccines. Mutations and the emergence of novel variants [93, 94] along with safety concerns [94, 95] are among the challenges ahead of the available vaccines. Hence, efforts to arrive at more effective vaccine [96] and therapeutics [97] are ongoing. Remdesivir is an FDA-approved antiviral drug expected to be active against emerging variants; however, limited data is available [98]. Recently, host-virus interplay mapping revealed that ELC exhibits antiviral activity against SARS-CoV-2 [99]. In this research, ELC decreased the infection near the cells that had received remdesivir treatment or uninfected cells [99]. It was attributed to targeting HSPA1A and increased HSP70 expression upon SARS-CoV-2 infection [99].

### Other copper-associated disorders

Copper is a critical micronutrient, which is necessary for the functionality of numerous enzymes. Cytochrome *c* oxidase (CcO) is among these enzymes, which is the final enzyme in the mitochondrial respiratory chain. Inherited loss-of-function mutations could occur in multiple genes including the ones, which encode proteins such as COA6 and SCO2 that are responsible for copper delivery to CcO. These mutations could lead to reduced CcO activity and severe pathologic conditions. On the other hand, copper has inherent toxicity due to its capability to produce hydroxyl radicals in biological systems, which limits direct copper supplementation for patients. Hence, the identification of efficient copper-transporting pharmacological agents seems to be inevitable [79]. Soma et al*.* used a candidate-based approach to investigate the ELC application to treat human disorders of copper metabolism. Attained results indicated that ELC could increase copper concentration within mitochondria and restore CcO activity. This approach could overcome the respiratory defects of COA6-deficient yeast cells. ELC restored copper-containing subunits of CcO in the copper-deficient zebrafish model and several copper-deficient mammalian cells, such as patient-derived cells with SCO2 mutations. ELC could mimic the missing copper transporters and chaperones to restore intracellular copper homeostasis [79].

#### Hypertrophic scar formation

Hypertrophic scars (HSs) enhance proliferation and reduce apoptosis in myofibroblasts. These cells are the main effector cells for dermal fibrosis. ELC has been utilized to stimulate myofibroblast apoptosis as a potential cure for HS [100]. In vitro studies showed that ELC managed to target myofibroblasts and inhibit their contractility as revealed by 4′,6-diamidino-2-phenylindole staining, and collagen gel contraction assay, respectively. Furthermore, extreme intracellular levels of Caspase-3, ROS, and cytochrome *c* proteins were determined by western blot analysis and flow cytometry. An increased number of apoptotic myofibroblasts upon ELC treatment was observed using immunofluorescence assays for α-smooth muscle actin and TUNEL. A previous study demonstrated a significantly lower scar elevation index in the ELC group compared with the control group. In light of these observations, ELC might be considered a candidate therapeutic approach for myofibroblast-related diseases including HS via augmentation of oxidative stress.

#### Menkes disease

**Fig. 4:**
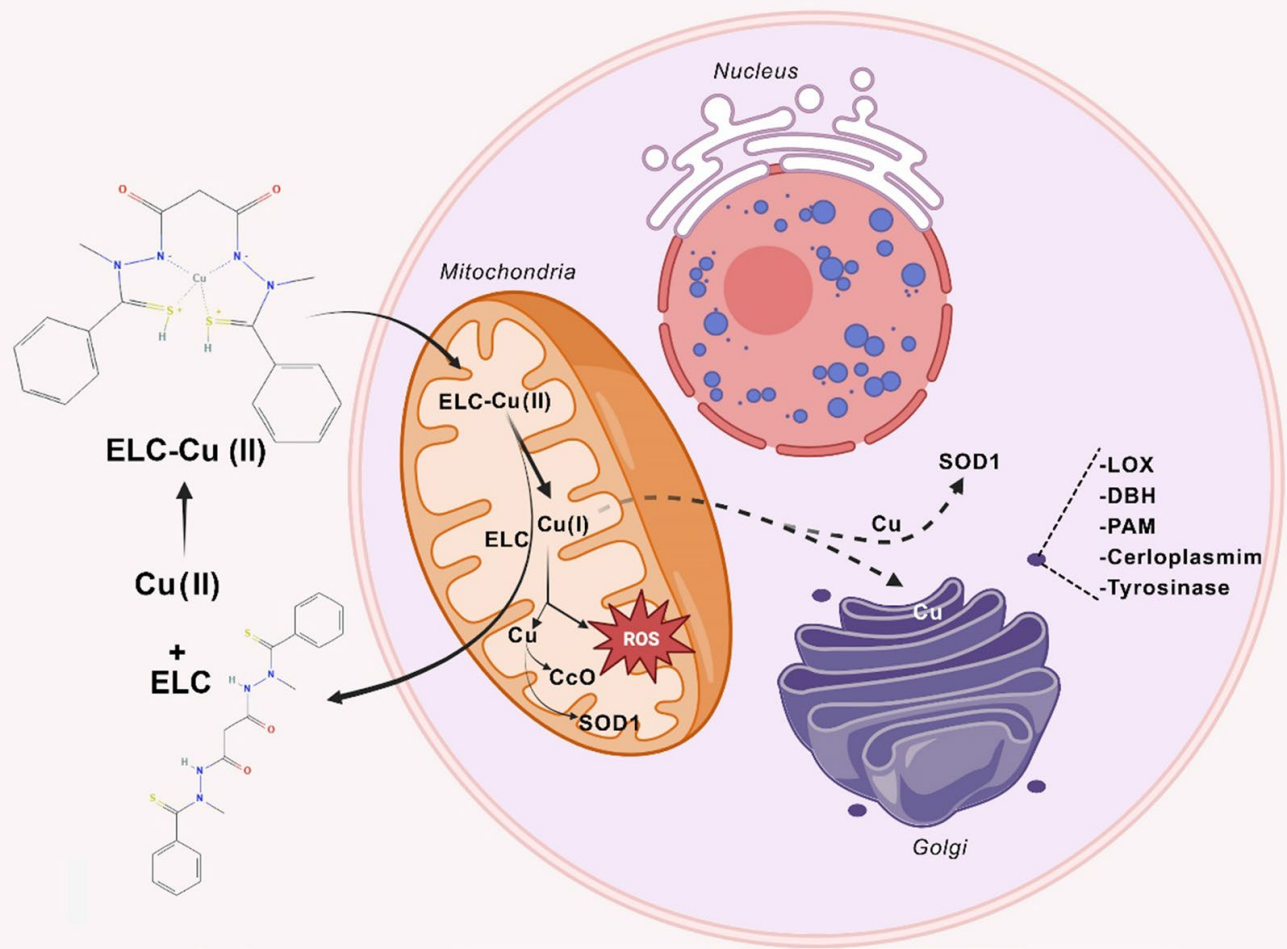
Copper delivery into the cell by ELC. In the extracellular environment, ELC binds copper (Cu^2+^) and transports it into mitochondria. Ferredoxin 1 (FDX1) is likely responsible for reducing the ELC-Cu(II) complex in the mitochondrial matrix into ELC and Cu(I). Reactive oxygen species (ROS) can be generated as a result of the release of Cu(I). The metalation of cytochrome *c* oxidase (CcO) with copper(I) has been demonstrated to be bioavailable. Other subcellular compartments may also receive copper(I) for use in cuproenzyme maturation. Including superoxide dismutase 1 (SOD1) in the mitochondria and cytosol and lysyl oxidase (LOX), dopamine β-hydroxylase (DBH), ceruloplasmin, peptidylglycine α [1] amidating monooxygenase (PAM), and tyrosinase in the secretory pathway

In Menkes pathology, as a fatal hereditary copper-deficiency disorder, ELC escorted the Cu to cupro-enzymes. Loss-of-function mutations in the ATP7A copper transporter have led to multiple pathologies in Menkes disease, such as disturbed energy production due to cytochrome *c* oxidase impairment in the mitochondria [101]. Guthrie et al*.* have suggested ELC for Menkes treatment and related disorders of hereditary copper deficiency via escorting copper to the mitochondria and increasing brain-levels of cytochrome *c* oxidase (Fig. 4). Their results indicated that ELC could prevent damage of neurodegenerative alterations and enhance the survival rate of mottled-brindled mouse, a murine model of severe Menkes disease [102]. Thus, ELC could be repurposed as a drug to treat this copper-related metabolic disorder [11].

## Advances in drug delivery of ELC

**Fig. 5:**
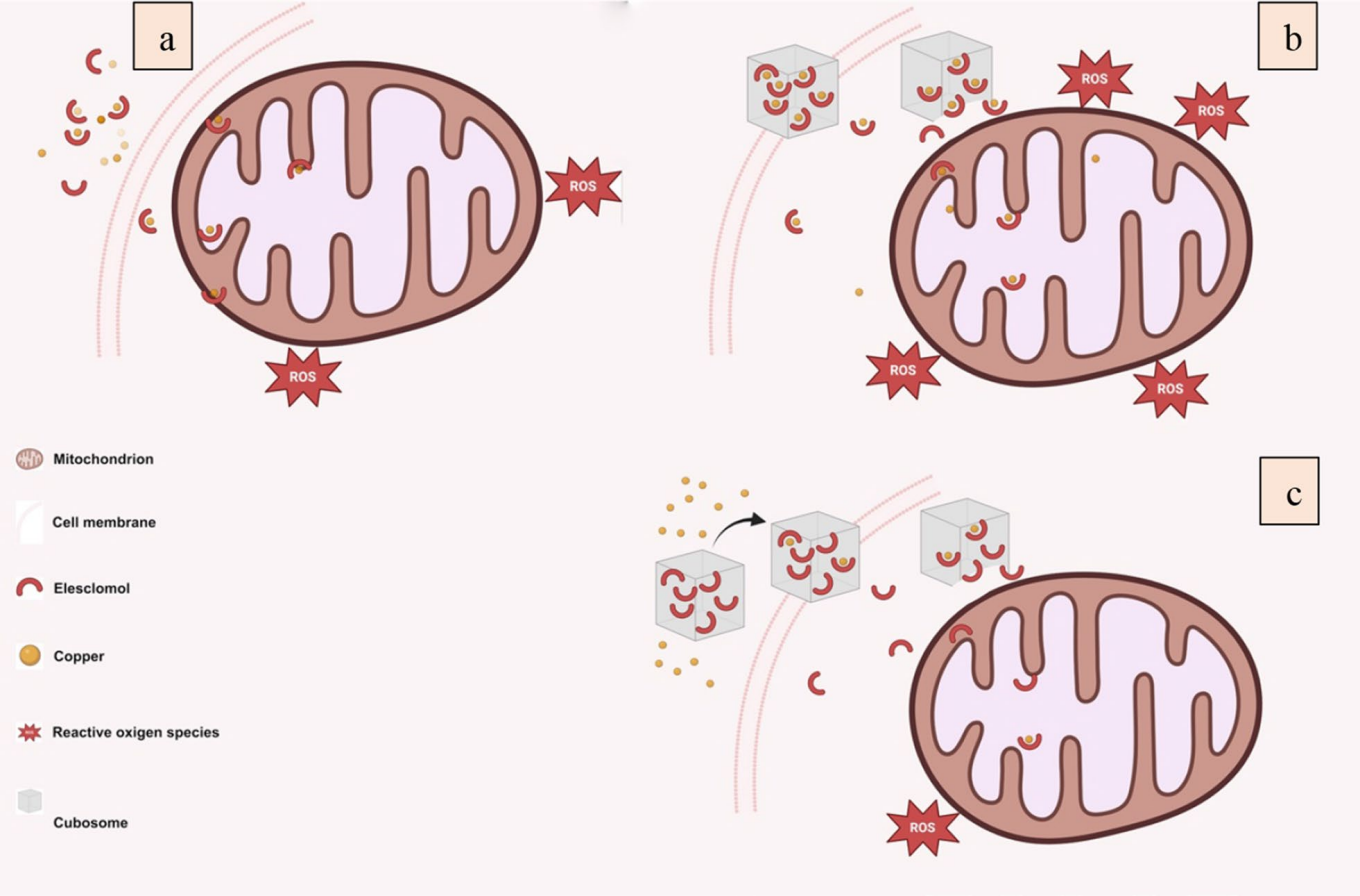
Delivery of ELC. **a** Free ELC binds to copper and enters the cells to increase the ROS. **b** Cubosomes loaded with the preformed ELC-Cu complex (ELC-Cu@Cub) improve cytotoxicity. **c** Cubosomes loaded only with ELC and copper added to the cell culture medium (ELC@Cub + Cu) limit the generation of ROS

Faria et al. developed a novel nano-carrier, which showed optimistic properties for systemic ELC-Cu administration. ELC, which has poor water-solubility, was encapsulated in monoolein-based cubosomes and then stabilized with Pluronic F127. The label-free time-lapse multi-photon fluorescence lifetime imaging microscopy (MP-FLIM) was used to track the NAD(P)H cofactors with sub-cellular resolution in live cells treated with this nano-carrier (Fig. 5). In vitro*,* cytotoxicity was higher than free drug when equal copper content and cubosomes were used. The encapsulation of ELC inside the cubosome decreases the cytotoxicity and restricts its binding to the copper present in the medium. To address this issue, pre-complexing of ELC with copper (before loading) could be effective. This strategy could end with the release of functional ELC-Cu complex in mitochondria proximity, where ROS is produced. Ultimately, this could lead to better anticancer activity [103].

A recent study has discussed the use of a ROS-sensitive biodegradable polymer called PHPM to load ELC and Cu into nanoparticles (NP@ELCCu) for cancer therapy. Once the NP@ELCCu is internalized by cancer cells, ELC and Cu are immediately released due to the excessive intracellular ROS. The concerted action of ELC and Cu could lead to the eradication of cancer cells through cuproptosis and provoking immune response. This study has aimed to address the limitations of existing immunotherapeutic agents and enhance the effects of cancer therapy by induction of cuproptosis and immune responses. The authors also have explored the changes in transcriptome of cancer cells treated with NP@ELCCu and its combination with anti-programmed cell death protein ligand-1 antibody (αPD-L1). The efficacy of NP@ELCCu in transporting Cu and inducing cuproptosis was evaluated in vitro, and the impact of NP@ELCCu on the transcriptome of cancer cells was analyzed using RNASeq. Furthermore, the administration of NP@ELCCu in a mouse model with subcutaneous bladder cancer was shown to trigger cuproptosis and remodel the tumor microenvironment. NP@ELCCu was also complemented with αPD-L1 antibody to enhance its therapeutic potential [104].

## Challenges and limitations

ELC has shown promise as a mitochondrion-targeted chemotherapeutic agent for cancer treatment. However, there are several key challenges in using ELC as a single agent for cancer therapy. High concentrations of ELC can induce mitochondrial toxicity by increasing mitochondrial superoxide levels and dissipation of mitochondrial membrane potential [20]. Moreover, ELC acts as a direct uncoupler of oxidative phosphorylation and a generalized inhibitor of electron transport activity in isolated mitochondria, which can inhibit bioenergetic functions [105]. In addition, the anticancer activity of ELC is dependent on its copper complexes, and the synthesis and characterization of these complexes could be challenging [29]. These limitations highlight the need for further research and optimizations to fully understand and overcome the challenges raised by ELC as a single agent for cancer treatment.

The efficacy of ELC can be improved by combining it with other anti-cancer agents. Additionally, ELC can be loaded with copper to form ELC-Cu complexes, which show higher cytotoxicity compared to the free drug. Improving the sensitivity of cancer cells to ELC represents another impressive approach. A combination of drugs could be considered a promising strategy to improve the sensitivity of cancer cells to ELC. The reduction of glycolytic activity can enhance cancer cell sensitivity to ELC. The activation or intensification of cancer cells' mitochondrial metabolism through hypoxia-inducible factor-1 α (HIF-1 α) and pyruvate dehydrogenase kinase-3 (PDK) (by nuclear accumbens-1 and dichloroacetate (DCA)) reduce the glycolysis of cancer cells. When used in combination with DCA, ELC has a synergistic inhibitory effect on tumors, thus increasing cancer cell sensitivity towards ELC. Moreover, this combination provides novel clinical insights for using ELC. However, the safety and efficacy of this combination require further verification through additional experiments. The investigation of ELC selectivity towards cancer cells represents a promising avenue for future research. The use of molecules that require activation to release ELC holds significant value in this regard. Of note, cancer cells exhibit a distinct profile in the production of ROS in comparison to normal cells. The susceptibility of cancer cells to ROS is heightened upon surpassing the cell threshold level. To enhance the selectivity of ELC towards cancer cells, molecules activated by the sensitivity of cancer cells to ROS may be utilized. This approach may result in a more discerning strategy that mitigates the typical side effects associated with metal-binding compounds. Additionally, the use of targeted delivery systems, in combination with the aforementioned approach, holds a great promise. The cytotoxicity of ELC in various mammalian cells warrants further investigations. Certain studies indicate that the cytotoxicity of ELC appears to be selective towards cancer cells. Peripheral blood mononuclear cells (PBMCs) in human remain unaffected at concentrations that have a significant killing effect on cancer cells. Additionally, ELC was found to be incapable of inducing copper ion enrichment in PBMCs. Nevertheless, some studies have suggested that normal mammalian cells experience certain effects on mitochondrial function as a result of being exposed to ELC. For instance, the treatment of CV-1 cells with over 40 μM ELC was discovered to increase the production of ROS in mitochondria, while significantly decreasing the mitochondrial membrane potential. According to existing reports, nearly one thousand patients have received high doses of ELC in clinical trials. The favorable tolerance of ELC by patients is a shared attribute of these clinical trials. For example, a phase I trial demonstrated that patients with solid tumors could tolerate up to 438 mg/m^2^ of ELC. No instances have been documented of patients encountering organic or functional impairment related to ELC. Hence, treatment with ELC presents a commendable safety profile, however, the safety of ELC merits additional scrutiny (reviewed in reference [66]).

Considering the concerns elucidated above, the convergence of artificial intelligence (AI) and nano-oncology emerges as a prospective next-generation approach. This is attributed to AI's substantial capability for swiftly analyzing a diverse array of factors linked to both patients and nano-candidate platforms. In greater details, AI algorithms hold the potential to aid in optimizing the characteristics of nanoparticles in alignment with the tumor microenvironment (TME). Furthermore, they can predict interactions between nanoparticles and the immune system, thereby surmounting biological obstacles, projecting cancer progression, assessing pharmacological profiles, and elevating the responsiveness to precision medicine therapies.

While the path ahead to address the limited translational success of nanoparticles in clinical therapies may be lengthy, the trajectory of nano-oncology is anticipated to revolutionize the landscape of cancer treatment. This transformative endeavor is anchored in the central goal of cancer nanomedicine: to enhance patient survival. This optimistic aspiration is expected to materialize shortly.

Addressing the challenges posed by ELC as a standalone cancer treatment requires a multifaceted approach that leverages both innovative strategies and existing scientific insights. One effective avenue is to explore combination therapies that enhance the efficacy of ELC. Combining ELC with other well-established anticancer agents can potentially synergize their effects and improve treatment outcomes. Furthermore, the formulation of ELC-loaded copper complexes demonstrates enhanced cytotoxicity, suggesting a promising route for overcoming its limitations. Additionally, strategies to sensitize cancer cells to ELC effects hold considerable potential. Modulating cancer cell metabolism by targeting glycolytic pathways or mitochondrial functions can heighten the sensitivity of cancer cells to ELC, making it a more potent treatment option.

The pursuit of selective targeting is another promising direction. Harnessing the distinct susceptibility of cancer cells to ROS offers a tailored approach to delivering ELC selectively to cancer cells. Molecules that release ELC in response to cancer-specific ROS levels could mitigate off-target effects. Implementing targeted delivery systems in conjunction with this approach can further enhance the specificity and effectiveness of ELC treatment.

The encouraging safety profile of ELC observed in clinical trials underscores its potential as a therapeutic agent. However, continued research is crucial to thoroughly understand its safety, especially in prolonged treatment regimens. Moreover, the integration of cutting-edge technologies such as artificial intelligence and nano-oncology presents an exciting opportunity. AI can guide the design of ELC-based therapies, predict interactions within the tumor microenvironment, and optimize treatment strategies for personalized medicine.

Ultimately, overcoming the limitations of ELC as a standalone agent requires a holistic approach that combines therapeutic innovations, advanced technologies, and an in-depth understanding of cancer biology. By embracing these strategies, the translation of ELC into effective clinical therapies can be advanced, contributing to the future of cancer treatment. In conclusion, the utilization of advanced methodologies, including computational techniques, bioinformatics tools, appropriate animal models, and finely tuned treatment strategies, holds the promise of crafting an efficacious model centered around nanocarriers for the prospective therapeutics of cancer using ELC.

## Conclusion and perspective

ELC is a unique mitochondrion-targeting agent, which induces oxidative stress and generates ROS beyond sustainable levels in cancer cells. Thus, it can trigger selective cell death. In a recent study, cuproptosis has been discovered and nominated as a novel mechanism of cell death for ELC. In the present review, the discovery, synthesis, mechanism of action, and clinical applications of ELC and its complexes were summarized. The main clinical applications introduced for ELC are as follows: (I) used as a ‘Trojan-horse’ fashion delivery, it transports copper molecules in copper deficiencies; (II) it has been employed effectively in various multi-drug resistant cancers; and (III) it has been used in pathways related to ROS generation. Despite the reported great achievements of ELC, it is noteworthy that the efficacy of ELC for eradication of tumors is affected by different factors when used as a single agent. It seems that cuproptosis could address questions that remained from previous studies. A combination of ELC with therapeutic agents is also an alternative way to augment its anticancer potency. Overall, considering the selectivity and pivotal roles of ELC, it can be considered an effective anti-cancer agent; however, further investigations are required before its proper clinical applications.

## Data Availability

Not applicable to this article as no new data were created or analysed in this study.

## Abbreviations

BITC: Benzyl isothiocyanate
CRPC: Castration-resistant prostate cancer
CGMP: Current good manufacturing practice
DCC: Dicyclohexylcarbodiimide
DEGs: Differentially expressed genes
EGCG: Epigallocate-3-gallate
ELC: Elesclomol
ETC: Electron transport chain
EwS: Ewing sarcoma
GC: Gastric cancer
HS: Hypertrophic scar
IKK: IκB kinase
JNK: Jun N-terminal kinase
LDH: Lactate dehydrogenase
MAP: Mitogen-activated protein
MDR: Multidrug resistance
MP-FLIM: Multiphoton-Fluorescence lifetime imaging microscopy
Mtb: Mycobacterium tuberculosis
NAC: N-acetylcysteine
NF-κB: Nuclear factor kappa B
OC: Ovarian cancer
PI3K: Phosphatinositide-3-kinase
RGES: Reverse gene expression score
ROS: Reactive oxygen species
SAR: Structure–activity relationship
TIC: Tumor initiating cell
TOS: Total oxidant status
UCP2: Uncoupling protein 2
